# Role of F-box Protein Cdc4 in Fungal Virulence and Sexual Reproduction of *Cryptococcus neoformans*

**DOI:** 10.3389/fcimb.2021.806465

**Published:** 2022-01-11

**Authors:** Ting Wu, Cheng-Li Fan, Lian-Tao Han, Yuan-Bing Guo, Tong-Bao Liu

**Affiliations:** ^1^ State Key Laboratory of Silkworm Genomic Biology, Southwest University, Chongqing, China; ^2^ Chongqing Key Laboratory of Microsporidia Infection and Control, Southwest University, Chongqing, China; ^3^ College of Animal Science and Technology, Southwest University, Chongqing, China; ^4^ Medical Research Institute, Southwest University, Chongqing, China

**Keywords:** *Cryptococcus neoformans*, F-box protein, Cdc4, sexual reproduction, virulence

## Abstract

*Cryptococcus neoformans* is an opportunistic yeast-like pathogen that mainly infects immunocompromised individuals and causes fatal meningitis. Sexual reproduction can promote the exchange of genetic material between different strains of *C. neoformans*, which is one of the reasons leading to the emergence of highly pathogenic and drug-resistant strains of *C. neoformans*. Although much research has been done on the regulation mechanism of *Cryptococcus* sexual reproduction, there are few studies on the sexual reproduction regulation of *Cryptococcus* by the ubiquitin-proteasome system. This study identified an F-box protein, Cdc4, which contains a putative F-box domain and eight WD40 domains. The expression pattern analysis showed that the *CDC4* gene was expressed in various developmental stages of *C. neoformans*, and the Cdc4 protein was localized in the nucleus of cryptococcal cells. *In vitro* stress responses assays showed that the *CDC4* overexpression strains are sensitive to SDS and MMS but not Congo red, implying that Cdc4 may regulate the cell membrane integrity and repair of DNA damage of *C. neoformans*. Fungal virulence assay showed that although the *cdc4*Δ mutant grows normally and can produce typical virulence factors such as capsule and melanin, the *cdc4*Δ mutant completely loses its pathogenicity in a mouse systemic-infection model. Fungal mating assays showed that Cdc4 is also essential for fungal sexual reproduction in *C. neoformans*. Although normal mating hyphae were observed during mating, the basidiospores’ production was blocked in bilateral mating between *cdc4*Δ mutants. Fungal nuclei development assay showed that the nuclei failed to undergo meiosis after fusion inside the basidia during the bilateral mating of *cdc4*Δ mutants, indicating that Cdc4 is critical to regulating meiosis during cryptococcal mating. In summary, our study revealed that the F-box protein Cdc4 is critical for fungal virulence and sexual reproduction in *C. neoformans*.

## Introduction

*Cryptococcus neoformans* is an encapsulated yeast pathogen that can infect the central nervous system (CNS) to cause fatal fungal meningitis in immunocompromised patients, resulting in hundreds of thousands of deaths each year (Casadevall and Perfect, 1998; Park et al., 2009; Rajasingham et al., 2017). In recent years, with the increase of the immune-deficient population, the infection of *Cryptococcus* is calling for more attention (Brown et al., 2012; May et al., 2016). As a human fungal pathogen, *C. neoformans* expresses several well-characterized virulence factors, including capsule formation, melanin production, and growth at mammalian body temperature (37°C), which favors the infection and the pathogenesis of *C. neoformans* (Zaragoza, 2019). *C. neoformans* is a heterothallic basidiomycetous fungus with two mating types, ⍺ and **a**, and can undergo a dimorphic transition to filamentous growth by mating and monokaryotic fruiting (Lin and Heitman, 2006). Besides its medical importance, *C. neoformans* has also been emerged as a model organism to study fungal genetics and pathogenesis.

The ubiquitin-proteasome system (UPS) is the major pathway of intracellular protein degradation, playing an important role in regulating a variety of cellular functions (Nandi et al., 2006; Liu and Xue, 2011). The UPS consists of ubiquitin, ubiquitin-activating enzyme E1, ubiquitin-conjugating enzyme E2s, ubiquitin ligase E3s, 26S proteasome, and deubiquitinates (DUBs) (Fang and Weissman, 2004; Ernst et al., 2013). The SCF (Skp1, Cullins, and F-box proteins) E3 ubiquitin ligases are among the best-understood groups of E3 ligases, in which the F-box protein is responsible for specific recognition and ubiquitination of protein substrates (Liu and Xue, 2011).

F-box protein contains an F-box domain of about 50 amino acids, which was initially found in human cycle protein F (Bai et al., 1994). F-box proteins are found in all eukaryotes and play an important role in the regulation of cell functions such as cell cycle, circadian clocks, nutrient sensing, and signal transduction (Jonkers and Rep, 2009). Meanwhile, F-box proteins also play a role in many pathologies such as sleep and mood disorders, diabetes, Parkinson’s disease, and bacterial and viral infections (Nguyen and Busino, 2020). The first fungal F-box protein to be identified and well-studied was the glucose repression resistant 1 (Grr1) in *S. cerevisiae* (Flick and Johnston, 1991; Jonkers and Rep, 2009). It has been shown to involve cell cycle regulation, nutritional sensing, and fungal morphogenesis by regulating its downstream target proteins (Flick and Johnston, 1991; Bai et al., 1996; Bernard and Andre, 2001; Blondel et al., 2005). Grr1 homologs such as Grr1 in *Candida albicans* (Butler et al., 2006), GrrA in *Aspergillus aspergillus* (Krappmann et al., 2006), and the Fbp1 in *Gibberella zeae* (Han et al., 2007) have also been functionally studied. Another F-box protein, Cdc4, has also been studied in *S. cerevisiae* (Feldman et al., 1997; Orlicky et al., 2003) and *C. albicans* (Atir-Lande et al., 2005; Shieh et al., 2005), which have also been shown to be associated with the cell cycle and morphological development of fungi. The involvement of the F-box proteins in the virulence of plant-pathogenic fungi has also been reported recently (Duyvesteijn et al., 2005; Han et al., 2007; Shi et al., 2019; Lim and Lee, 2020). So far, the role of F-box protein in the virulence of human fungal pathogens is rarely reported.

Our previous studies identified an F-box protein Fbp1 that is essential for fungal virulence in *C. neoformans* (Liu et al., 2011; Liu and Xue, 2014). However, genomic sequence analysis showed that *C. neoformans* contains at least 19 F-box proteins (**Table 1**), among which the rest have not been studied except Fbp1, and their functions are still unknown. In this study, we identified the second F-box protein, Cdc4, in *C. neoformans* and showed that Cdc4 is essential for fungal virulence and sexual reproduction in *C. neoformans*.

**Table 1:** F-box proteins in *C. neoformans*.

Gene ID	Name	Homolog in *S. cerevisiae*	E value	Function	References
CNAG_00134				hypothetical protein	
CNAG_00416	Fwd1	Cdc4	9e-16	F-box/WD-repeat protein lin-23	
CNAG_00693	Cdc4	Cdc4	7e-100	F-box and WD-40 domain-containing protein Cdc4	(Feldman et al., 1997)
CNAG_03157				hypothetical protein	
CNAG_03421				hypothetical protein	
CNAG_04341	Pof6	Rcy1	2e-59	f-box protein pof6, recyclin-1	(Wiederkehr et al., 2000)
CNAG_04462	Fbp9	Hrt3	4e-17	F-box protein 9	
CNAG_04606				hypothetical protein	
CNAG_05280	Fbp1	Grr1	1e-68	ubiquitin-protein ligase, F-box and leucine-rich repeat protein Grr1	(Bailey and Woodword, 1984; Liu et al., 2011)
CNAG_05294	Fbw7	Cdc4	4e-43	ubiquitin-protein ligase, F-box and WD-40 domain-containing protein Cdc4	(Goh and Surana, 1999)
CNAG_05450				hypothetical protein	
CNAG_05454				hypothetical protein	
CNAG_05773	Met30	Met30	8e-72	F-box and WD-40 domain-containing protein Met30	(Thomas et al., 1995)
CNAG_05874				hypothetical protein	
CNAG_06382	Fwd2	Tup1	1e-11	beta-transducin repeat containing	(Keleher et al., 1992)
CNAG_06722				hypothetical protein	
CNAG_07482	Saf1			SCF-associated factor 1	
CNAG_07551		Met30	0.001	hypothetical protein	
CNAG_07702				hypothetical protein	

## Materials and Methods

### Strains and Growth Conditions

The *C. neoformans* strains used in this study are listed in **Table S1**. The *Cryptococcus* strains were conventionally grown at 30°C on YPD medium. MS medium (Murashige and Skoog medium) and V8 medium were used for mating and sporulation assays as described previously (Fan et al., 2019). A diluted Sabouraud medium was used for capsule formation induction of *Cryptococcus* strains (Zaragoza and Casadevall, 2004). The other media used in this study were prepared as described previously (Liu et al., 2011).

### *CDC4* Gene Expression Assay

To detect the expression of the *CDC4* gene during the mating process of *C. neoformans*, we measured the *CDC4* expression at the mRNA levels throughout the mating process, using quantitative real-time PCR (qRT-PCR). The preparation and collection of the mating mixtures, total RNA extraction, cDNA synthesis, and the qRT-PCR operation were performed as described previously (Fan et al., 2019). The gene expression level of *CDC4* was normalized by the internal control gene *ACTIN*, and the relative expression level of *CDC4* was measured by the comparative threshold cycle (C_T_) method (Livak and Schmittgen, 2001).

The expression of *CDC4* was also examined by constructing a fusion expression strain of the *CDC4* gene native promoter and mCherry. A 1.5-Kb promoter fragment of the *CDC4* gene was amplified and cloned into pTBL3 to generate the *CDC4* promoter and mCherry fusion plasmid pTBL83. To determine the subcellular localization of Cdc4 in *C. neoformans*, we amplified the coding region of *CDC4* from H99 genomic DNA using primers TL170/TL171 and cloned it into pCN19 to generate the expression vector pTBL39 of the GFP-Cdc4 fusion protein. The resulting plasmids, pTBL83 and pTBL39, were linearized by *Sal*I and *Xmn*I, respectively, and biolistically transformed into the wild-type strains (H99 and KN99**a**) or *cdc4*Δ mutants as described previously (Davidson et al., 2000). Stable transformants were further confirmed by screening in YPD medium containing nourseothricin sulfate (100 mg/liter) and fluorescence observation with a confocal laser microscope (Olympus, FV1200).

### DAPI Staining

To monitor the fungal nuclei positioning in *Cryptococcus* cells, we performed a DAPI staining as previously described with minor modification (Bahn et al., 2005; Liu et al., 2011). Briefly, cell cultures of each *Cryptococcus* strain were washed twice with 1 × PBS buffer and fixed with 9.3% formaldehyde for 10 min. The fixed cells were washed twice with 1 × PBS buffer and permeabilized for 5 min with an equal volume of 1 × PBS buffer containing 1% Triton X-100. Next, the cells were washed twice with 1 × PBS buffer and resuspended in 1 × PBS buffer. Then equal volumes of DAPI mixture (20 ng/ml DAPI, 1 mg/mL antifade, 40% glycerol) and cell suspension were mixed and observed with a confocal laser microscope (Olympus, FV1200).

### Generation of *cdc4*Δ Mutants and Their Complemented Strains

The *CDC4* gene was knocked out in both mating types of the wild-type strains (H99 and KN99**a**) using the split marker strategy, as described previously (Kim et al., 2009; Fan et al., 2019). The primers used for *CDC4* gene knockout are shown in **Table S2**. The *cdc4*Δ mutants were further confirmed by diagnostic PCR using positive primers F4/R4 (TL42 and TL59) and negative primers F3/R3 (TL40 and TL41) and Southern blot analysis. To complement the *CDC4* gene in *cdc4*Δ mutants, we amplified a 3.9-Kb genomic DNA fragment containing the promoter, ORF, and terminator sequences of the *CDC4* gene using primers TL157/TL158 and cloned it into the pTBL1 vector (Fan et al., 2019) to generate the *CDC4* gene complementary vector pTBL119. The resulting vector, pTBL119, was linearized by *Xmn*I and biolistically transformed in both ⍺ and **a** mating-type *cdc4*Δ mutant strains. Mating assays were used to identify transformants that complemented the *cdc4*Δ mutant phenotype.

To generate the *CDC4* overexpression *CDC4* ^OE^ strains, we examined the *CDC4* gene expression in the GFP-Cdc4 fusion protein strain constructed above using qRT-PCR. After confirming the overexpression of the *CDC4* gene, the GFP-Cdc4 fusion protein strain was used as *CDC4* overexpression strain for subsequent experiments.

### Assays for Melanin and Capsule Production

To evaluate the role of the F-box protein Cdc4 in melanin production in *C. neoformans*, we induced the yeast cells of the wild-type H99, *cdc4*Δ mutant, *cdc4*Δ::*CDC4*, or *CDC4* ^OE^ strains on Niger seed agar medium as described previously (Fan et al., 2019). The agar plates were incubated at 30°C or 37°C for 2 days to evaluate the pigmentation of the fungal colonies. To examine the capsule production, a total of 10^6^ cells from the overnight cultures of each cryptococcal strain were induced in a diluted Sabouraud medium as described previously (Zaragoza and Casadevall, 2004; Fan and Liu, 2021). The capsule size was analyzed as described previously (Liu et al., 2011).

### Virulence Studies

Overnight cultures in YPD broth of each *Cryptococcus* strain were washed twice with the 1 × PBS buffer and then resuspended at a final concentration of 2 ×10^6^ cells/ml. Ten female C57 BL/6 mice (Chongqing Medical University, China) per group were infected intranasally with 1 × 10^5^ yeast cells of the wild-type H99, *cdc4*Δ mutant, *cdc4*Δ::*CDC4*, or *CDC4* ^OE^ strain as described previously (Cox et al., 2000). Over the course of the animal experiment, mice that appeared moribund or in pain were sacrificed by CO_2_ inhalation. Mice that survived to 80 days postinfection (dpi) without exhibiting signs of disease were also sacrificed to terminate the survival assay. Survival data from the animal experiments were statistically analyzed between paired groups using the log-rank test with PRISM version 8.0 (GraphPad Software, San Diego, CA) (*P* values of <0.001 were considered significant).

### Histopathology and Fungal Burdens in Infected Organs

According to an animal protocol approved by Southwest University, the infected mice were sacrificed at the endpoint of the animal experiment. Infected tissues (brains, lungs, and spleens) were isolated from the mice infected by each *Cryptococcus* strain at the endpoint of the animal experiments were fixed in 10% formalin solution and sent to Servicebio (Servicebio, Wuhan, China) for section preparation. Tissue slides were stained with hematoxylin and eosin (H&E) or methenamine silver and observed by light microscopy. Infected brains, lungs, and spleens were also dissected and homogenized in 1 × PBS buffer using a tissue homogenizer. The homogenates were diluted, and 100 microliters of each diluent were spread on the YPD plates containing ampicillin and chloramphenicol for CFU enumeration.

### Assays of *Cryptococcus*-Macrophage Interaction and Serum Treatment

*Cryptococcus*-macrophage interaction assay was performed as described previously (Liu et al., 2011; Liu and Xue, 2014). Briefly, a total of 2 × 10^5^ yeast cells washed twice with 1 × PBS and opsonized with 20% mouse complement were added to each well containing J774 macrophage-like cells. After 2 h of incubation, nonadherent extracellular yeast cells were removed by washing with fresh DMEM, and the cultures were incubated for another 0, 2, or 22 h. At the indicated time points, distilled water was added to each well to lyse macrophage cells after removing the DMEM. The lysate was spread on YPD plates, and yeast CFU counts were used to determine the phagocytosis rate and intracellular proliferation.

Serum treatment and cell viability assay of each *Cryptococcus* strain was performed as described previously (Fan and Liu, 2021). The serum used in this study is the mouse serum (M5905, Sigma). Aliquots were taken out at the indicated time points and plated to YPD medium after serial dilution to determine cell viability.

### Fungal Mating Assays

To examine the role of Cdc4 in fungal mating, overnight cultured yeast cells of the opposite mating type were mixed in equal amounts after washing with 1 × PBS buffer and cultured on MS or V8 medium at 25 degrees in the dark. To monitor nuclear positioning in *cdc4*Δ mutants during mating, a *NOP1-mCherry-NAT* cassette (Fan et al., 2019) was transformed biolistically into the *cdc4*Δ mutants of both ⍺ and **a** mating types. The resulting strains, TBL153 and TBL144, were mixed and cultured on MS to induce fungal mating. Mating hyphae and basidiospore formation were examined and imaged by photography using the Olympus CX41 light microscope after incubation for 14 days.

## Results

### Identification of *CDC4* Gene in *C. neoformans*

Our previous study identified an F-box protein Fbp1 in *C. neoformans*, and functional analysis revealed that Fbp1 plays an essential role in sexual reproduction and virulence in *C. neoformans* (Liu et al., 2011; Liu and Xue, 2014). Given that the F-box protein Fbp1 plays an important role in *C. neoformans*, we then scanned the genome sequence of *C. neoformans* strain H99 and identified 19 proteins (**Table 1**) that contain the F-box domain in the H99 genome database (https://fungidb.org/fungidb/app/record/dataset/DS_8f0322af73). Among them, six proteins contain WD40 repeats, and in this study, we selected one of them, Cdc4 (CNAG_00693), for further functional study.

**Figure 1:**
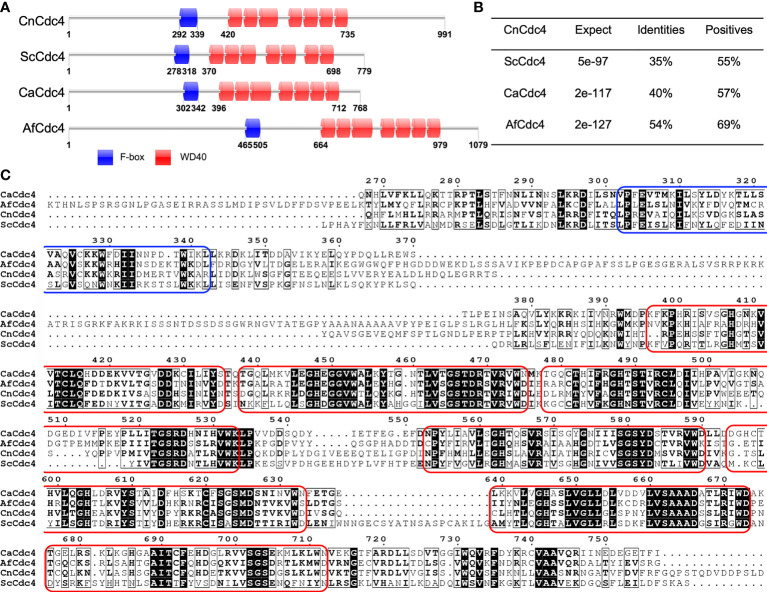
Sequence analysis of Cdc4 proteins. **(A)** Schematic illustration of Cdc4 proteins in *C. neoformans* (CnCdc4), *S. cerevisiae* (ScCdc4), *C. albicans* (CaCdc4), and *A. fumigatus* (AfCdc4). F-box, F-box domain; WD, WD40 domains. **(B)** *C. neoformans* Cdc4 protein shows high identity and similarity to the Cdc4 proteins of *S. cerevisiae*, *C. albicans*, and *A. fumigatus*. **(C)** Comparison of the region spanning F-box and WD40 domains in Cdc4 protein sequences of *C. neoformans* (CnCdc4), *S. cerevisiae* (ScCdc4), *C. albicans* (CaCdc4), and *A. fumigatus* (AfCdc4). The F-box domain and each WD40 domain were labeled by blue and red round rectangular box, respectively.

Cdc4 contains 991 amino acids with an F-box domain and a WD-40 domain containing seven WD40 repeats (**Figures 1A, C**). Cdc4 shows high sequence similarity to several Cdc4 proteins reported in fungi, including Cdc4 in *S. cerevisiae* (ScCdc4), *C. albicans* (CaCdc4), and *A. fumigatus* (AfCdc4) (**Figure 1B**). In *S. cerevisiae*, the Cdc4 is required for the mitotic cell cycle and meiotic nuclear division (Simchen and Hirschberg, 1977; Goh and Surana, 1999). The Cdc4 protein was also proven to be critical for filamentous growth in *C. albicans* (Shieh et al., 2005). *Cryptococcus* Cdc4 shows high sequence identity and sequence similarity with Cdc4s in *S. cerevisiae*, *C. albicans*, and *A. fumigatus*, suggesting it may play an important role in *C. neoformans*. Due to the importance of the cell cycle and its regulation in both the development and pathogenesis of *C. neoformans*, we decided to investigate the function of Cdc4.

### *CDC4* Expression Pattern Analyses

**Figure 2:**
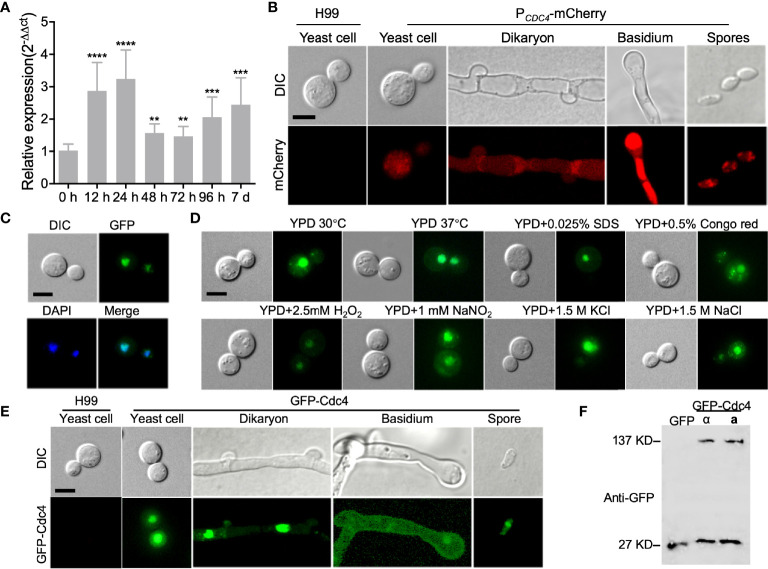
Expression pattern of *CDC4* in *C. neoformans*. **(A)** Expression of *CDC4* during mating on V8 medium was detected by qRT-PCR. Mating mixtures of the wild-type strains were collected at indicated times and used for RNA extraction and cDNA synthesis. The comparative cycle threshold (C_T_) method was used to quantify the relative expression of the *CDC4* gene, and the *ACTIN* gene was used as an endogenous reference. The experiment was repeated three times. **P < 0.01. ***P < 0.001. ****P < 0.0001. **(B)** The expression of P*_CDC4_*-mCherry of *C. neoformans* at different developmental stages was examined by confocal microscopy. Typical images of the bright-field and fluorescence of the yeast cells, dikaryon, basidium, and spores are shown. Bar, 5 µm. **(C)** Localization of Cdc4 in *C. neoformans*. Yeast cells of the GFP-Cdc4 expressing strain were stained with DAPI and examined by confocal microscopy. GFP-Cdc4 is localized in the nucleus of *Cryptococcus* cells. Bar, 5 µm. **(D)** Localization of GFP-Cdc4 in yeast cells under different stresses. The cells were observed by confocal microscopy. Bar, 5 µm. **(E)** Localization of GFP-Cdc4 at different developmental stages of *Cryptococcus* mating. The yeast cells, dikaryon, basidium, and spores of the GFP-Cdc4 expressing strain were observed by confocal microscopy. Bar, 5 µm. **(F)** Detection of GFP-Cdc4 fusion protein in *C. neoformans.* Total proteins from the *Cryptococcus* strains (⍺ and **a** mating types) expressing the GFP-Cdc4 fusion proteins were used to detect the expression of GFP-Cdc4 in a Western blot with anti-GFP antibodies. The *Cryptococcus* strain expressing GFP was used as a control.

To examine the expression of *CDC4* during the different developmental stages of *C. neoformans*, we first analyzed the *CDC4* expression using qRT-PCR. After incubation for 0, 12, 24, 48, 72, 96 h, and 7 d on V8 plates, mating mixtures between H99 and KN99**a** were collected and used for RNA extraction and cDNA synthesis. Our results showed that, compared to expression at the 0-h time point, the expression of *CDC4* was up-regulated during mating, reaching a peak at 24 hours after mating (**Figure 2A**). This result indicates that the *CDC4* gene may play an important role in the mating process of *C. neoformans*.

To detect the *CDC4* expression more intuitively during mating, we also analyzed the temporal control of *CDC4* expression by constructing and expressing the *CDC4* native promoter-mCherry fusion *P_CDC4_-mCherry* in *C. neoformans*. The expression of mCherry was observed in yeast cells of the *P_CDC4_-mCherry* strains (**Figure 2B**). In addition, the expression of mCherry was also detected in the dikaryotic hyphae, basidia, and basidiospores of *P_CDC4_-mCherry* strains, indicating that the *CDC4* gene is expressed in different developmental stages in *C. neoformans* (**Figure 2B**).

### Cdc4 Localization in *C. neoformans*

To examine the subcellular localization of Cdc4 in *C. neoformans*, the *GFP-CDC4* fusion construct (pTBL39) was linearized with *Xmn*I and transformed into *cdc4*Δ mutant strains of both α and **a** mating types. Fluorescence observation revealed that the GFP-Cdc4 fusion proteins are likely located in the nucleus of the yeast cells (**Figure 2C**). The subcellular localization of GFP-Cdc4 fusion protein was further confirmed DAPI staining of yeast cells (**Figure 2C**). Furthermore, we tested the localization of GFP-Cdc4 inside the yeast cell under different stress conditions such as high-temperature stress (37°C), oxidative stress (2.5 mM H_2_O_2_), osmotic stress (1.5M KCl and 1.5M NaCl), cell wall stress (0.025% SDS and 0.5% Congo red), or nitrosative stress (1mM NaNO_2_, pH=4.0) and different developmental stages and found that Cdc4 does have subcellular localization in both cell membrane and cell nucleus (**Figures 2D, E**). However, in addition to a strong fluorescence signal in the nucleus of *Cryptococcus* cells, a weak fluorescence signal was also observed in the cytoplasm of the GFP-Cdc4 strain. We then detected the stability of GFP-Cdc4 fusion protein using Western blot with anti-GFP antibody and found that part of the GFP-Cdc4 fusion protein was hydrolyzed (**Figure 2F**). We speculated that the weak GFP signal in the cytoplasm might be due to the hydrolysis of GFP-Cdc4 fusion protein rather than the proper localization of GFP-Cdc4.

### Cdc4 Regulates Cell Membrane Integrity and Repair of DNA Damage

**Figure 3:**
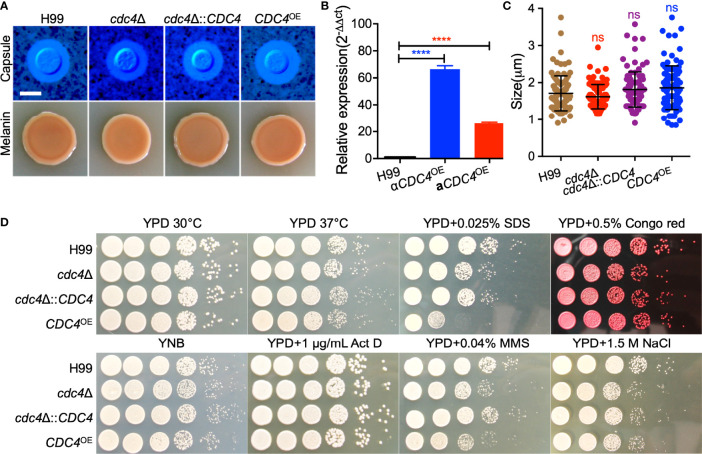
Cdc4 is not essential for virulence factor development but is required for cell membrane integrity and DNA damage repair. **(A)** Capsule formation (top) and melanin production (bottom) were assayed on DME medium and Niger seed medium, respectively. Capsule production of *Cryptococcus* strains was observed by India ink staining after grown on DME medium for three days at 37°C. Melanin levels produced by each *Cryptococcus* strain were imaged after incubation for 3 days at 37°C. Bar, 5 µm. **(B)** The overexpression of the *CDC4* gene in *C. neoformans* was measured by the relative qRT-PCR analysis. ****P < 0.0001. **(C)** Statistical analysis of the capsule formation in each *Cryptococcus* strain. ns, not significant. **(D)**. Growth of the *Cryptococcus* strains under different stress conditions for two days at 30°C. Act D, actinomycin D; MMS, methyl methanesulfonate. The name of the strain is shown on the left, and the condition is indicated on the top.

To investigate the function of Cdc4 protein in *C. neoformans*, we generated the *cdc4*Δ mutant and its complemented strain *cdc4*Δ::*CDC4* and *CDC4* overexpression strain *CDC4* ^OE^ (**Figure 3B**). Then we examined the development of virulence factors such as capsule and melanin production and growth at 37°C in the above-mentioned *Cryptococcus* strains *in vitro*. The above three strains produced regular capsules on DME medium, normal melanin on Niger seed agar, and showed normal growth at 37°C (**Figures 3D** and **S2**), suggesting that Cdc4 is not essential for the development of these virulence factors *in vitro* (**Figures 3A, C**).

Although Cdc4 is not involved in the development of several well-characterized virulence factors, we examined the growth of each *Cryptococcus* strain under stresses that mimic the hostile host environment. We tested the growth of *Cryptococcus* strain under osmotic stress (1.5 M NaCl), chemicals that target cell integrity (0.025% SDS, 0.5% Congo red), inhibitors of DNA transcription and replication (1 µg/mL Actinomycin D), and DNA-damaging agents (0.04% methyl methanesulfonate (MMS)). Our results showed that the cells of the *CDC4* overexpression strain were hypersensitive to 0.025% SDS, but not Congo red, indicating that the *CDC4* overexpression strain has cell membrane integrity defects (**Figure 3D**). In addition, both the *CDC4* knockout and overexpression strains were slightly sensitive to 0.04% MMS, but not to Actinomycin D, suggesting that Cdc4 protein may play a role in the repair of DNA damage (**Figure 3D**).

### Cdc4 Is Required for Fungal Infection

**Figure 4:**
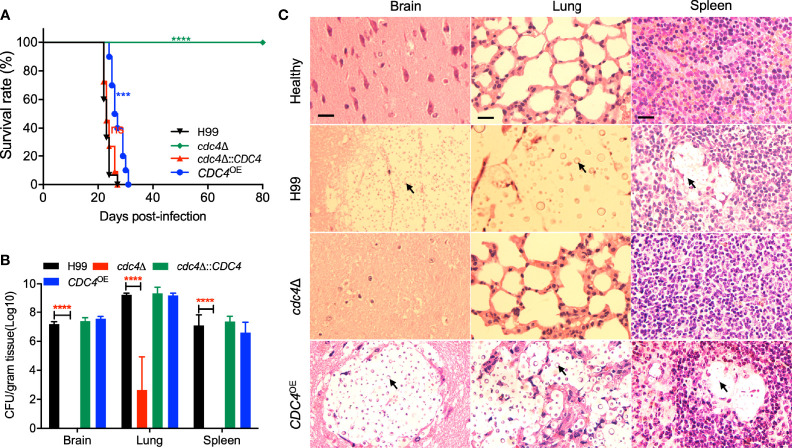
Cdc4 regulates virulence of *C. neoformans* in a mouse inhalation model of cryptococcosis. **(A)** Female C57 BL/6 mice were intranasally infected with 1 × 10^5^ cells of the wild type (H99), *cdc4*Δ mutant, *cdc4*Δ::*CDC4* complemented strain, and the *CDC4* overexpression *CDC4* ^OE^ strain. The *cdc4*Δ mutant is completely avirulent compared with the wild-type strain H99. ns, not significant. ***P < 0.001. ****P < 0.0001 (determined by the log-rank (Mantel–Cox) test). **(B)** Brains, lungs, and spleens of five mice infected with H99, the *cdc4*Δ mutant, the *cdc4*Δ::*CDC4* complemented strain, and the *CDC4* overexpression *CDC4* ^OE^ strain were isolated at the end time point of the infection. Homogenates of the organs were prepared for colony-forming unit (CFU) counting. The data shown are the mean ± SD for values of five mice. ****P ≤ 0.0001 (determined by Mann-Whitney test). **(C)** H&E-stained slides from the cross-sections of infected organs were prepared at the endpoint of the infection and imaged by microscopy. The yeast cells are indicated by arrows. Bars, 20 µm.

Because fungal virulence is a complex trait, we next examined the virulence of *cdc4*Δ mutant in a murine inhalation model of systemic *C. neoformans* infection, although Cdc4 is not involved in the formation of virulence factors. Consistent with our previous studies, the mice infected by the wild-type strain H99 were terminated at 22-27 dpi due to lethal infection. Surprisingly, the *cdc4*Δ mutant tested is completely avirulent, and the mice infected by the *cdc4*Δ mutant remained healthy and continued to gain weight even after 80 dpi (**Figure 4A**). The mice infected by the complemented strain of the *cdc4*Δ mutant survived between 22 to 27 dpi, which was not different from that of the wild-type strain, indicating that the avirulent phenotype in the *cdc4*Δ mutant is caused by the disruption of the *CDC4* gene. Interestingly, the *CDC4* overexpression *CDC4* ^OE^ strain also showed a significant virulence attenuation when compared with the wild-type strain, and the mice infected with the *CDC4* ^OE^ strain survived between 24 and 31 dpi (*P* < 0.0001) (**Figure 4A**).

To examine why the *cdc4*Δ mutant has a virulence defect, we evaluated the fungal burdens of tissues of infected mice at the endpoint of the animal study. Brains, lungs, and spleens from three to five mice infected by each *Cryptococcus* strain were isolated, and CFU per gram fresh organ was calculated to determine the fungal burdens. Our results showed that 10^6^, 10^9^, and 10^6^ CFU were recovered from the brains, lungs, and spleens of the mice infected by the wild-type strain and sacrificed at the endpoint of the animal experiment. Mice infected by the *cdc4*Δ mutant were sacrificed at 80 dpi, and mouse tissues were isolated to determine the fungal burden. Interestingly, no yeast cells were recovered from the brains and spleens infected by the *cdc4*Δ mutant, and only ~10^3^ yeast CFU on average were recovered in each gram of lung (**Figure 4B**). Fungal lesion development in tissues infected with each *Cryptococcus* strain at the endpoint of the animal experiment was also observed in H&E-stained slides. As shown in **Figure 4C**, both the wild type and the *CDC4* overexpression strain caused severe damage in the infected lungs, with abundant yeast cells containing big capsules. In contrast, only very limited damage was produced in the lungs infected by the *cdc4*Δ mutant, and very few yeast cells were observed. After infection with the wild-type H99 and the *CDC4* overexpression strain, severe tissue damage with visible lesion development was visualized in both brains and spleens at the endpoint of the animal experiment. However, no detectable damage or lesion was detected in brains and spleens infected by the *cdc4*Δ mutant even at 80 dpi (**Figure 4C**).

### Cdc4 Is Essential for Progression of Fungal Infection

**Figure 5:**
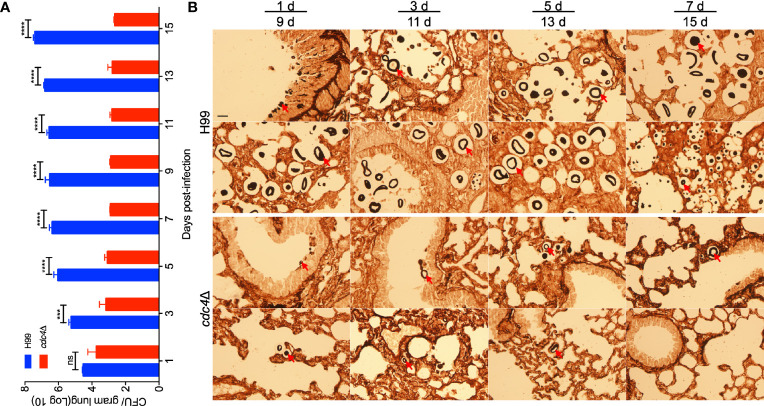
Progression of *cdc4*Δ mutant infection in lungs of infected mice. Lungs of the mice infected by *cdc4*Δ mutant and the wild type were isolated at 1, 3, 5, 7, 9,11, 13, and 15 days after infection. The number of CFU was measured in lung homogenates **(A)**. The data shown are the mean ± SD for values of five mice. ns, not significant. ***P < 0.001. ****P < 0.0001 (determined by Mann-Whitney test). **(B)** Methenamine silver-stained slides were also prepared from the cross-section of the lung and observed by light microscopy. The yeast cells are indicated by arrows. Bar, 20 µm.

To better understand the role of Cdc4 during the infection progression, we examined the fungal burdens in lungs infected by wild-type H99 and *cdc4*Δ mutant at 1, 3, 5, 7, 9, 11, 13, and 15 dpi and visualized the development of fungal lesions in Methenamine silver-stained slides. CFU counts showed that the number of yeast cells in lungs infected by the wild-type strain increased gradually with the extension of time after inoculation, while that of the *cdc4*Δ mutant-infected mice decreased and remained at a low but persistent level (~10^3^) (**Figure 5A**). Histopathology results indicated that the *cdc4*Δ mutant could not cause infection in the lungs, and a few yeast cells could be seen in the slides, while the intensive accumulation of cryptococcal cells and lesion development were shown in the lungs infected by the wild-type strain (**Figure 5B**). These results suggested that Cdc4 is essential for the development of cryptococcosis in a murine inhalation model.

### Cdc4 Is Important for Proliferation Inside Macrophages and Survival in the Host Complement System

**Figure 6:**
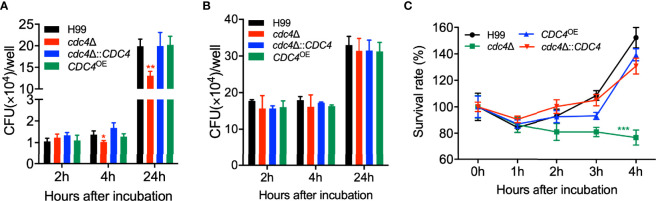
Cdc4 plays an important role in the proliferation inside the macrophage and the survival in the host complement system. **(A)** The proliferation of *Cryptococcus* inside the macrophage was performed using the J774 murine macrophage cells. After incubation of indicated time, the lysates of the macrophage cultures were spread on YPD plates, and the CFU counts were used to measure the intracellular proliferation and macrophage killing. *P < 0.05; **P < 0.01 (determined by Mann-Whitney test). As a control, the growth of the wild type and the *cdc4*Δ mutant was also measured in DMEM without macrophages **(B)**. **(C)** Overnight cultures of the same strains as in **(A)** were mixed with mouse serum and incubated at 37 °C for indicated times. One hundred microliters of the dilute (10^3^ dilution) was spread on YPD plates, and the CFU counts were used to measure the *Cryptococcus* cell viability. ns, not significant; ***P < 0.001 (determined by Mann-Whitney test).

Our virulence study showed that the *cdc4*Δ mutant is avirulent and remains at a low but persistent level in infected lungs in a murine model of systemic infection. Thus, we hypothesized that the *cdc4*Δ mutant might have a defect in proliferation in host macrophages, and extracellular cryptococcal cells may not survive in the hostile environment of the host. To verify our hypotheses, we first performed a *Cryptococcus*-macrophage interaction assay using the murine J774 macrophage-like cells. After two hours of coincubation of fungal cells and macrophages, the CFU counts recovered from the *cdc4*Δ mutant-coincubated macrophages were comparable to those recovered from macrophages coincubated with the wild-type strain, indicating a similar phagocytosis level between the *cdc4*Δ mutant and the wild-type strain (**Figure 6A**). However, after 4 or 24 hours of coincubation, the CFU counts recovered from the *cdc4*Δ mutant-coincubated macrophages were significantly fewer than that of the wild-type-interacting macrophages (*P* < 0.05 and *P* < 0.01, respectively, **Figure 6A**). Meanwhile, we also tested the fungal growth rate in DMEM without macrophages and YNB medium, and we found that both the *cdc4*Δ mutant and the wild type had similar growth in these media (**Figures 6B**, **S2**). These results suggested that the *cdc4*Δ mutant proliferates slower than the wild-type strain once it is engulfed by macrophages, which could be one of the reasons for the significant virulence attenuation of the *cdc4*Δ mutant in the mouse systemic infection model. Then we examined the viability of fungal cells incubated with serum for 1, 2, 3, and 4 h to verify whether components of the host complement system could damage *Cryptococcus* cells. The CFU counts showed that the survival rate of the *cdc4*Δ mutant was significantly lower than that of the wild type after 4 h coincubation with mouse serum (*P* < 0.001, **Figure 6C**), indicating that the components of the host complement system did have more severe damage on *cdc4*Δ mutant, which could be another reason for the significant virulence defect of the *cdc4*Δ mutant in the mouse systemic infection model. Due to the difference in intracellular growth between the *cdc4*Δ mutant and the wild-type strain, we concluded that Cdc4 plays a critical role in the proliferation of fungal cells in macrophages.

### Cdc4 Is Essential for Sexual Reproduction

**Figure 7:**
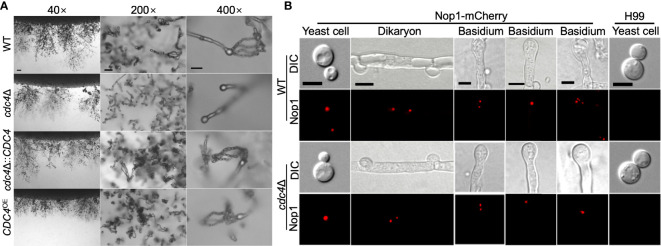
Cdc4 is essential for sexual reproduction in *C. neoformans*. **(A)** Mating assays for the bilateral mating of the wild type, *cdc4*Δ mutants, *cdc4*Δ::*CDC4* strains, and *CDC4* ^OE^ strains were performed on MS medium. Mating structures at ×40 magnification (left, bar = 100 µm), ×200 magnification (middle, bar = 50 µm), and ×400 magnification (right, bar = 10 µm) were photographed after 14 days of incubation in the dark at 25°C. **(B)** Fungal nuclei development in yeast cells, mating hyphae, and basidia of wild type and *cdc4*Δ mutants. The mating cultures were isolated and visualized by confocal microscopy after incubation on MS medium in the dark for 7 or 14 days. DIC, differential interference contrast; Nop1, Nop1-mCherry. WT, wild-type strains H99 and KN99**a**. Bars, 5 μm.

*C. neoformans* is a basidiomycete with two mating types (α and **a**) and can undergo heterothallic sexual reproduction to produce mating hyphae and basidiospores. To evaluate the role of Cdc4 in fungal mating, we generated the *cdc4*Δ mutants in both H99 and KN99**a** strain backgrounds. The development of dikaryotic hyphae and basidiospores was examined in bilateral mating in *cdc4*Δ mutants. Like the wild-type strains, the *cdc4*Δ mutants produced normal mating hyphae. However, the bilateral mating between *cdc4*Δ mutants failed to produce basidiospores, indicating that Cdc4 is essential for sporulation in *C. neoformans* (**Figure 7A**).

To explore why the *cdc4*Δ mutants are unable to produce spores, we constructed a fusion protein of the Nop1 nucleolar protein and mCherry (C-terminus tagged) and introduced it into the native *NOP1* gene in both mating type of the wild type and *cdc4*Δ mutant strains to image the fungal nuclei development at different stages of cryptococcal sexual reproduction. α and **a** mating types of the wild type or *cdc4*Δ mutants expressing the Nop1-mCherry were crossed, respectively, and their nuclear development was monitored during the mating process. As shown in **Figure 7B**, a single nucleus was observed in each yeast cell of both the wild type and the *cdc4*Δ mutant, while two separated nuclei can be visualized in each dikaryotic hypha produced after cell fusion.

During mating, both the wild type and the *cdc4*Δ mutants had a single fused nucleus in the young basidium, showing that both strains undergo normal nuclear fusion to produce basidia, which is consistent with previous findings (Liu et al., 2011; Fan et al., 2019). However, nuclei in the bilateral mating of *cdc4*Δ mutants failed to undergo meiosis after fusion, and only a single nucleus could be identified in each mature basidium after 14 days of incubation, whereas all basidia produced four nuclei in wild-type mating (**Figure 7B**).

These findings suggested that Cdc4 is required for the regulation of meiosis during mating, which may help explain why the *cdc4*Δ mutants were unable to generate spores when mating with a partner in bilateral mating. Nevertheless, when cultured in rich media, the *cdc4*Δ mutants had a normal growth rate as well as normal nuclear division, indicating that Cdc4 is not involved in the cell cycle during mitotic division. Our findings, taken together, suggest that the F-box protein Cdc4 may be involved only in the regulation of meiosis during the mating phase.

## Disscussion

F-box proteins are the key components of the SCF E3 ubiquitin ligase responsible for specific recognition and ubiquitination of downstream substrates, thereby ensuring specific degradation of substrates by the UPS. F-box proteins are found in all eukaryotes and are involved in a large variety of cellular processes, and their importance has been gradually recognized. In this study, we identified and functionally characterized the second F-box protein Cdc4, a homolog of the *Saccharomyces* Cdc4, in *C. neoformans*. Analysis of expression pattern showed that the *CDC4* gene was expressed in various developmental stages of *C. neoformans*, and the Cdc4 protein was localized on the nucleus and cell membrane of *Cryptococcus* cells. *CDC4* overexpression but not deletion resulted in the SDS sensitivity of *Cryptococcus*, suggesting that Cdc4 regulates the integrity of the cell membrane of *Cryptococcus*. Our results further showed that Cdc4 is critical for fungal virulence and sexual reproduction in *C. neoformans*, similar to the function of another F-box protein, Fbp1, identified by us previously. Since F-box proteins have also been reported to play roles in fungal infectivity in plant-pathogenic fungi (Duyvesteijn et al., 2005; Han et al., 2007; Shi et al., 2019; Lim and Lee, 2020)., the involvement of F-box proteins in virulence is likely conserved in fungal pathogens.

*Cryptococcus* Cdc4 shares sequence and structural similarity with Cdc4 in *S. cerevisiae*, *C. albicans*, and *A. fumigatus* (**Figures 1A, B**). In *S. cerevisiae*, Cdc4 is a cell division cycle protein that controls the cell cycle and regulates pseudohyphal growth (Chou et al., 2004; Jonkers and Rep, 2009). In *C. albicans*, Cdc4 plays a role in the switch from hyphal to yeast-like growth (Atir-Lande et al., 2005; Shieh et al., 2005), and two proteins Sol1 and Pcl5 have been identified as substrates of Cdc4 (Atir-Lande et al., 2005; Simon et al., 2013). However, Cdc4 does not regulate cell morphology in *C. neoformans*, as *cdc4*Δ mutants produced normal yeast cells when grown in liquid medium. In *S. cerevisiae*, Cdc4 regulates filamentous growth by degrading transcription factor Tec1 (Chou et al., 2004). Another F-box protein in *S. cerevisiae*, Grr1, positively regulates glucose sensing by targeting Mth1 and Std1 to dissociate the binding of Rgt1 with promoters of hexose transporters. However, we could not find the obvious sequence homolog for either Mth1, Std1, or Rgt1 in the genomes of *C. neoformans* (Liu et al., 2011). Such results may indicate that the downstream substrates of Grr1 protein may be different in the two species. It is still unknown whether *Cryptococcus* has similar substrates, so identifying downstream regulatory substrates of Cdc4 will help explain why Cdc4 is different in the regulation of morphological development between the two species.

Protein subcellular localization in our study showed that the *Cryptococcus* GFP-Cdc4 fusion protein localized in the nucleus of *Cryptococcus* cells (**Figure 2**), which is consistent with the nuclear localization of the Cdc4 protein in *S. cerevisiae* (Blondel et al., 2000). However, in addition to a strong fluorescence signal in the nucleus of *Cryptococcus* cells, a weak fluorescence signal was also observed in the cytoplasm of the GFP-Cdc4 strain.

One possible reason is that the promoter used in the construction of the *GFP-CDC4* fusion expression vector is Histone 3, which is a strong promoter, leading to a large amount of GFP-Cdc4 fusion protein expression while excessive GFP-Cdc4 may lead to incorrect localization in the cell. Another possibility is that the GFP-Cdc4 fusion protein is partially hydrolyzed, leading to the release of the free GFP and hence the fluorescence signal in the cytoplasm of *Cryptococcus* cells. Our subsequent Western blot assay also proved that the GFP-Cdc4 fusion protein did undergo hydrolysis and release the free GFP protein. Thus, our results indicate that the *Cryptococcus* Cdc4 protein is located in the nucleus, while the weak fluorescence signal observed in the cytoplasm of the GFP-Cdc4 strain is due to the free GFP produced during the hydrolysis of the GFP-Cdc4 fusion protein.

So far, two F-box proteins, Fbp1 and Cdc4, which have been studied in *C. neoformans*, are both involved in the virulence and sexual reproduction process of *C. neoformans* (Liu et al., 2011; Liu and Xue, 2014). Fungal virulence assay showed that both the *fbp1*Δ mutant and *cdc4*Δ mutant could not disseminate to other organs such as brains and spleens following pulmonary infection in the murine inhalation model of cryptococcosis, suggesting that the block of dissemination of both mutants is due to their inability to leave the lung (Liu and Xue, 2014). Both the *fbp1*Δ mutant and *cdc4*Δ mutant showed a defect in intracellular proliferation after phagocytosis in a *Cryptococcus*-macrophage interaction assay, which likely contributes to their virulence attenuation in a murine inhalation model of cryptococcosis (Liu and Xue, 2014). The fungal mating assay showed that both Fbp1 and Cdc4 affect the sexual reproduction of *Cryptococcus* by regulating the nuclear division process of meiosis (Liu et al., 2011). Both Fbp1 and Cdc4 are involved in virulence and sexual reproduction in *C. neoformans* through similar mechanisms, so it would be interesting to explore how these two proteins affect the virulence and sexual reproduction of *Cryptococcus* by regulating their respective downstream substrates. Overall, our study identified another F-box protein that regulates virulence and sexual reproduction in *C. neoformans*, which may help to conclude that the F-box protein-mediated virulence and sexual reproduction regulation mechanism is likely conserved in fungi.

## Data Availability Statement

The datasets presented in this study can be found in online repositories. The names of the repository/repositories and accession number(s) can be found in the article/**Supplementary Material**.

## Ethics Statement

The animal studies conducted at Southwest University were in full compliance with “Guidelines on Ethical Treatment of Experimental Animals (2006, No. 398)” issued by the Ministry of Science and Technology of China and the “Regulation on the Management of Experimental Animals (2006, No. 195)” issued by Chongqing Municipal People’s Government. The Animal Ethics Committee of Southwest University approved all of the vertebrate studies.

## Author Contributions

T-BL conceived and designed the experiments, and wrote the manuscript. TW, C-LF, L-TH, and Y-BG performed the experiments and acquired the data. T-BL, TW, and C-LF analyze the data. T-BL obtained the funding. All authors reviewed the manuscript and approved it for publication.

## Funding

This work was supported by the National Natural Science Foundation of China (31970145 and 32170203) and Natural Science Foundation of Chongqing, China (cstc2021jcyj-msxmX1077).

## Conflict of Interest

The authors declare that the research was conducted in the absence of any commercial or financial relationships that could be construed as a potential conflict of interest.

## Publisher’s Note

All claims expressed in this article are solely those of the authors and do not necessarily represent those of their affiliated organizations, or those of the publisher, the editors and the reviewers. Any product that may be evaluated in this article, or claim that may be made by its manufacturer, is not guaranteed or endorsed by the publisher.
