# Supplementary material for: Role of F-box Protein Cdc4 in Fungal Virulence and Sexual Reproduction of Cryptococcus neoformans
Source: Front Cell Infect Microbiol. 2022 Jan 11;11:806465. doi: 10.3389/fcimb.2021.806465 (PMC8787122; doi:10.3389/fcimb.2021.806465)
Supplement: Supplementary file 1 [file DataSheet_1.docx]

Supplementary Figure S1


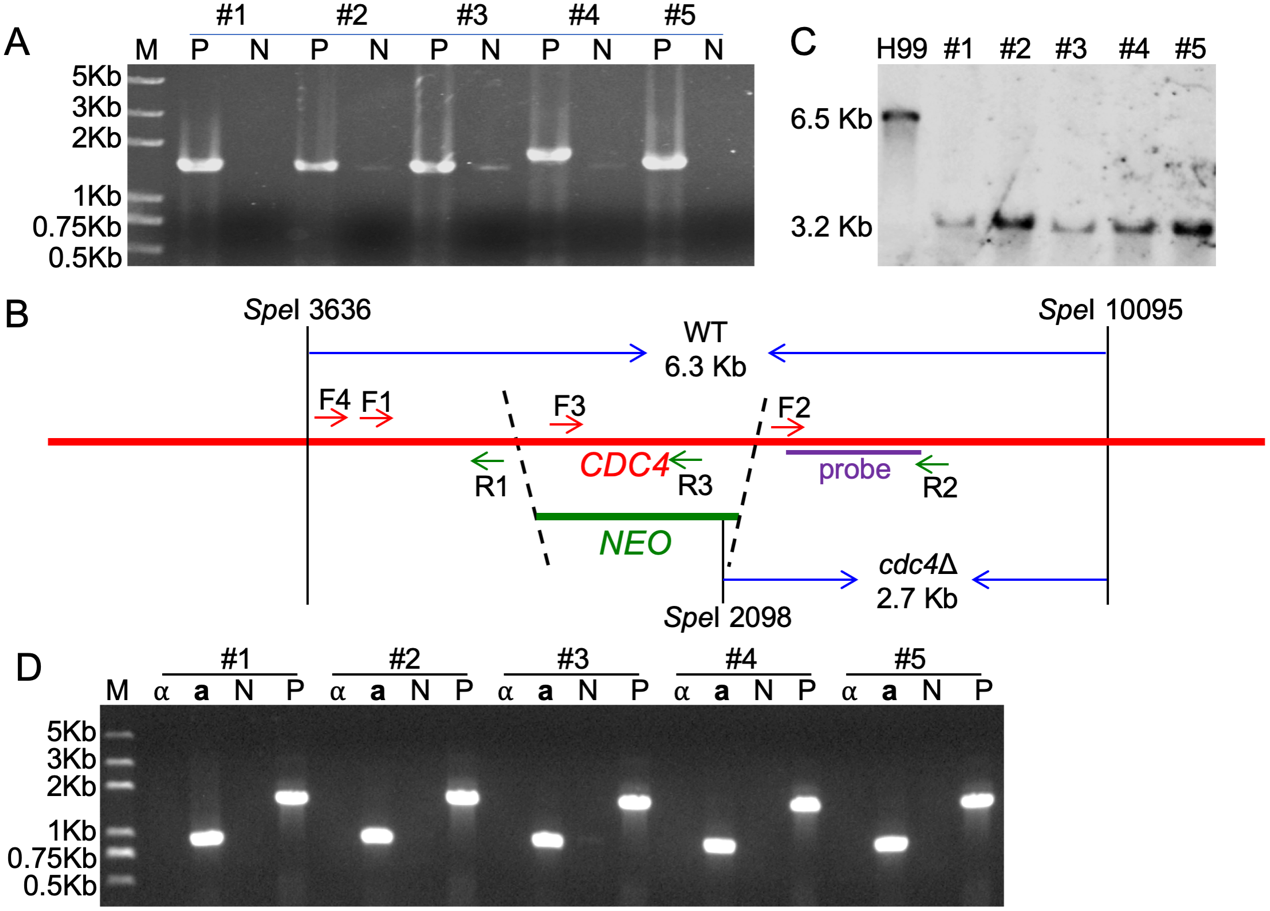


**FIGURE S1**. Generation of *cdc4*Δ mutants. (A) The diagnostic PCR validation of the five transformants with G418 resistance. P: positive primers, TL42/TL59 (F4/R4 in S1B); N: negative primers, TL40/TL41 (F3/R3 in S1B). (B) Restriction enzyme used to digest of the genomic DNAs in Southern blotting. The probe was synthesized using the PCR products amplified with TL382/TL39 (F2/R2) as templates. (C) Southern blotting analysis of the *CDC4* knockout mutants. A total amount of 50 μg of each genomic DNA was digested with *Spe*I, fractionated, and hybridized with a *CDC4* downstream flanking sequence-specific probe, as shown in S1B. As expected, a 6.5 and 3.2 Kb bands were generated in the wild-type strain H99 and the *cdc4*∆ mutants, respectively. (D) The diagnostic PCR validation of the **a** mating-type *cdc4*Δ mutants. ⍺: ⍺ mating-type specific primers, TL67/TL68; **a**: **a** mating-type specific primers, TL69/TL70. P: positive primers, TL42/TL59 (F4/R4 in S1B); N: negative primers, TL40/TL41 (F3/R3 in S1B).
