# Supplementary material for: Role of F-box Protein Cdc4 in Fungal Virulence and Sexual Reproduction of Cryptococcus neoformans
Source: Front Cell Infect Microbiol. 2022 Jan 11;11:806465. doi: 10.3389/fcimb.2021.806465 (PMC8787122; doi:10.3389/fcimb.2021.806465)
Supplement: Supplementary file 2 [file DataSheet_2.docx]

Supplementary Figure S2


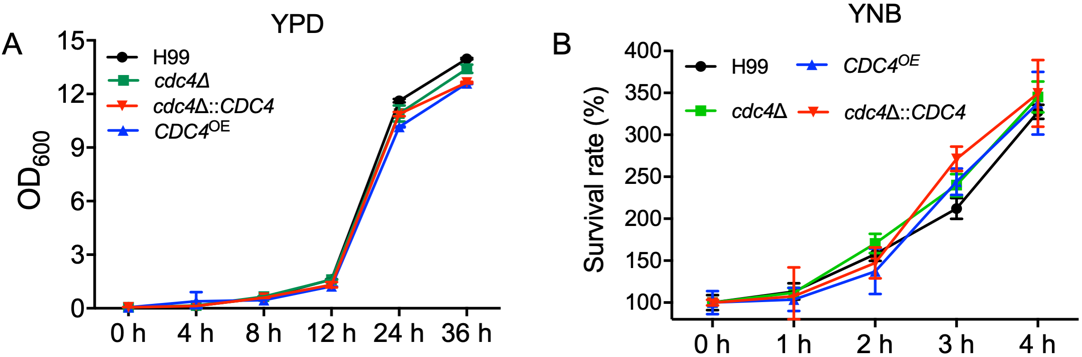


**FIGURE S2**. Growth of *cdc4*Δ mutants in YPD or YNB media. (A) Overnight cultures of each *Cryptococcus* strain were diluted in YPD broth to a final OD_600_ 0.05 and incubated with shaking at 200 rpm at 30°C. At indicated time points, 1 mL of the cultures were taken to measure the value of OD_600_. (B) Overnight cultures of each *Cryptococcus* strain were washed three times with ddH_2_O and then diluted in 500 µL YNB medium to a final concentration of 200 cells/mL and incubated with shaking at 200 rpm at 30°C. Aliquots were taken out and spread onto the YPD medium to determine cell viability after serial dilution at the indicated time points.
