# Supplementary material for: Role of F-box Protein Cdc4 in Fungal Virulence and Sexual Reproduction of Cryptococcus neoformans
Source: Front Cell Infect Microbiol. 2022 Jan 11;11:806465. doi: 10.3389/fcimb.2021.806465 (PMC8787122; doi:10.3389/fcimb.2021.806465)
Supplement: Supplementary file 3 [file Table_1.docx]

Table S1 Strains and plasmids used in this study

| Strains and plasmids | Genotype or properties | Source or reference |
| --- | --- | --- |
| *C. neoformans* | | |
| H99 | *MAT*α | (Perfect et al., 1993) |
| KN99**a** | *MAT***a** | (Nielsen et al., 2003) |
| TBL39 | *MAT*α *cdc4*Δ::*NEO* | In this study |
| TBL40 | *MAT***a** *cdc4*Δ::*NEO* | In this study |
| TBL41 | *MAT*α *cdc4*Δ::*NEO* *CDC4*::*NAT* | In this study |
| TBL42 | *MAT***a** *cdc4*Δ::*NEO* *CDC4*::*NAT* | In this study |
| TBL76 | *MAT*α *cdc4*Δ::*NEO* *GFP-CDC4:*:*NAT* | In this study |
| TBL77 | *MAT***a** *cdc4*Δ::*NEO* *GFP-CDC4*::*NAT* | In this study |
| TBL139 | *MAT*α *cdc4*Δ::*NEO* *NOP1-GFP*::*NAT* | In this study |
| TBL140 | *MAT***a** *cdc4*Δ::*NEO* *NOP1-GFP*::*NAT* | In this study |
| TBL144 | *MAT***a** *cdc4*Δ::*NEO* *NOP1-mCherry*::*NAT* | In this study |
| TBL153 | *MAT*α *cdc4*Δ::*NEO* *NOP1-mCherry*::*NAT* | In this study |
| TBL167 | *MAT*α P*_CDC4_*-mCherry::*NAT* | In this study |
| TBL168 | *MAT***a** P*_CDC4_*-mCherry::*NAT* | In this study |
| TBL192 | *MAT*α P*_H3_*-GFP::*NAT* | In this study |
| Plasmids | | |
| pCN19 | Amp^r^ Plasmid harboring *GFP* under histone H3 promoter | (Price et al., 2008) |
| pTBL1 | Amp^r^ Plasmid harboring *NAT* marker | (Fan et al., 2019) |
| pTBL3 | Amp^r^ Plasmid harboring *mCherry-GPD1* terminator and *NAT* marker | (Fan et al., 2019) |
| pTBL5 | Amp^r^ Plasmid harboring *P_ACTIN_*-mCherry-*GPD1* terminator and *NAT* marker | In this study |
| pTBL39 | Amp^r^ Vector for *P_H3_-GFP-CDC4* for Cdc4 localization | In this study |
| pTBL72 | Amp^r^ Vector for *P_ACTIN_-NOP1-mCherry-NAT* for nuclear positioning | (Fan et al., 2019) |
| pTBL79 | Amp^r^ Vector for *P_CDC4_-CDC4-NAT* for *CDC4* complementation | In this study |
| pTBL83 | Amp^r^ Vector for *P_CDC4_-mCherry-NAT* for temporal expression assay | In this study |
| pTBL119 | Amp^r^ Vector for *P_CDC4_-CDC4-NAT* for *CDC4* complementation | In this study |

Fan, C.L., Han, L.T., Jiang, S.T., Chang, A.N., Zhou, Z.Y., and Liu, T.B. (2019). The Cys2His2 zinc finger protein Zfp1 regulates sexual reproduction and virulence in *Cryptococcus neoformans*. *Fungal Genet Biol* 124**,** 59-72.

Nielsen, K., Cox, G.M., Wang, P., Toffaletti, D.L., Perfect, J.R., and Heitman, J. (2003). Sexual cycle of *Cryptococcus neoformans* var. *grubii* and virulence of congenic a and alpha isolates. *Infect Immun* 71**,** 4831-4841.

Perfect, J.R., Ketabchi, N., Cox, G.M., Ingram, C.W., and Beiser, C.L. (1993). Karyotyping of *Cryptococcus neoformans* as an epidemiological tool. *J Clin Microbiol* 31**,** 3305-3309.

Price, M.S., Nichols, C.B., and Alspaugh, J.A. (2008). The *Cryptococcus neoformans* Rho-GDP dissociation inhibitor mediates intracellular survival and virulence. *Infect Immun* 76**,** 5729-5737.
