# Supplementary material for: Role of F-box Protein Cdc4 in Fungal Virulence and Sexual Reproduction of Cryptococcus neoformans
Source: Front Cell Infect Microbiol. 2022 Jan 11;11:806465. doi: 10.3389/fcimb.2021.806465 (PMC8787122; doi:10.3389/fcimb.2021.806465)
Supplement: Supplementary file 4 [file Table_2.docx]

Table S2 PCR primers used in this study

| Primers | Targeted genes | Sequence (5’-3’) |
| --- | --- | --- |
| TL17 | M13F | GTAAAACGACGGCCAG |
| TL18 | M13R | CAGGAAACAGCTATGAC |
| TL19 | *NEO* split F | GGGCGCCCGGTTCTTTTTGTCA |
| TL20 | *NEO* split R | TTGGTGGTCGAATGGGCAGGTAGC |
| TL36 | *CDC4* KO F1 | CACAATGGGACGCTATATGACG |
| TL37 | *CDC4* KO R1 | CTGGCCGTCGTTTTACGTGTTGGGTGTGCCCTGCATC |
| TL38 | *CDC4* KO F2 | GTCATAGCTGTTTCCTGAGAGATGGAGGATGCTGATGAG |
| TL39 | *CDC4* KO R2 | CGTCCGGAAGTGGTCTCCTATC |
| TL40 | *CDC4* KO F3 | CTGTTAGGCGAAGTCCTCCTAG |
| TL41 | *CDC4* KO R3 | GAACCGCTGACAAGGGTGTGAC |
| TL42 | *CDC4* KO F4 | CACTCGCGTCGAGACAATCAG |
| TL59 | *NEO* R4 | TGTGGATGCTGGCGGAGGATA |
| TL67 | *STE20A* ⍺ F | CCAAAAGCTGATGCTGTGGA |
| TL68 | *STE20A* ⍺ R | AGGACATCTATAGCAGAT |
| TL69 | *STE20A* a F | TCCACTGGCAACCCTGCGAG |
| TL70 | *STE20A* a R | ATCAGAGACAGAGGAGCAAGAC |
| TL151 | Cdc4-mCherry F | CCCAACATGTCTGGATCCATGCAGGGCACACCCAACACAA(BamHI) |
| TL152 | Cdc4-mCherry R | TCTAGAACTAGTGGATCCATATGAATGCTCATCAGCATCC(BamHI) |
| TL157 | *CDC4* Comp F | GATATCGAATTCCTGCAGCCCGGGGGATCCGCGCGGATGTGGTCGTGA(BamHI) |
| TL158 | *CDC4* Comp R | CGGTGGCGGCCGCTCTAGAACTAGTGGATCGCAGGCGCGGAATGTAAAATG(BamHI) |
| TL170 | GFP-Cdc4 F | GACGAGCTGTAcGGATCCATGCAGGGCACACCCAACACAA |
| TL171 | GFP-Cdc4 R | CTGGCGGCCGTTACTAGTTCAATATGAATGCTCATCAGCA |
| TL467 | *CDC4* PRO F | ACGGTATCGATAAGCTTGCGCGGATGTGGTCGTGA(HindIII) |
| TL468 | *CDC4* PRO R | CTAGAACTAGTGGATCCCGCGTATGAATCTAGGCAAGCC(BamHI) |
| TL531 | *CDC4* QPCR F1 | TTCACGATCTCTCGGGTAGT |
| TL521 | *CDC4* QPCR R1 | GCATCCTCCATCTCTTCATCC |
| TL984 | *ACTIN* QPCR F1 | CGACAACGAGGGCCGATAGCAC |
| TL985 | *ACTIN* QPCR R1 | GAAGGCAGGGGCATTGAAAGTCTC |
